# Stand-alone MRI tool for semiautomatic volumetry of abdominal adipose compartments in patients with obesity

**DOI:** 10.1038/s41598-025-87578-4

**Published:** 2025-03-18

**Authors:** A. Linder, T. Eggebrecht, N. Linder, R. Stange, A. Schaudinn, M. Blüher, T. Denecke, Harald Busse

**Affiliations:** 1 Department of Diagnostic and Interventional Radiology, Leipzig University Hospital, Liebigstrasse 20 – Haus 4, 04103 Leipzig, Germany; 2 Integrated Research and Treatment Center (IFB) Adiposity Diseases, Leipzig University Medical Center, Leipzig, Germany; 3 Department of Radiology and Nuclear Medicine, Cantonal Hospital St. Gallen, St. Gallen, Switzerland; 4 Helmholtz Institute for Metabolic, Obesity and Vascular Research (HI-MAG) of the Helmholtz Zentrum München at the University of Leipzig and University Hospital Leipzig, Leipzig, Germany

**Keywords:** Obesity, Adipose tissue, Visceral fat, Subcutaneous fat, Quantification, Software tool, MRI, Biomarkers, Health care, Medical research

## Abstract

Abdominal adipose tissue (AT) amounts are increasingly considered as potential biomarkers for a variety of diseases and clinical questions, for instance, in diabetology, oncology or cardiovascular medicine. Despite the emergence of automated deep-learning methods for tissue quantification, interactive (supervised) segmentation tools will typically be used for model training. In comparison with CT-based approaches, MRI segmentation tools are more complex and less common. This work aims to validate a novel MRI-based tissue volumetry against a reference method in patients with (pre-) obesity. The new tool (segfatMR) was developed under a Matlab-based, open-source software framework and combines fast automatic pre-segmentation followed by manual (expert) corrections where needed. Analyses were performed retrospectively on a subset of clinical research MRI datasets (1.5 T Achieva XR, Philips Healthcare) and involved the segmentation of datasets from 20 patients (10 women/men) aged 25.1–63.1 (mean 48.5) years with BMIs between 28.3 and 58.8 (mean 36.8) kg/m^2^. Two independent expert readers analyzed the abdominopelvic data (30–40 slices, mean 35.8) with segfatMR and a widely used commercial tool (sliceOmatic). Coefficients of determination (*R*^2^), bias and limits of agreement (Bland Altman) were determined. Segmentation performance (*R*^2^ between methods) was excellent for both readers for SAT (> 0.99) and very high for VAT (around 0.90). The novel method was almost twice as fast as the reference standard – 25 and 19 s/slice (R1 and R2) vs. 40 and 34 s/slice. The presented semiautomatic segmentation tool enables a fast and accurate quantification of whole abdominopelvic adipose tissue volume in obesity studies. Use, adjustments and extensions of the MRI volumetry tool are facilitated by the open-source design on a standard PC.

## Introduction

Within a few decades only, obesity has become an epidemic with a rising prevalence in both adults and children^1^. The major side effects are a higher risk for metabolic syndrome, cardiovascular diseases, type-2-diabetes^2,3^, musculoskeletal^4,5^ and psychological disorders^6^ as well as cancer. The overall costs for treatment also pose a substantial socioeconomic burden^7^.

Excess weight in itself, however, is not necessarily the key factor for the development of the above diseases. Overweight may be subdivided into more and less ‘healthy’ forms in a metabolic context. Not all patients with overweight will present with a metabolic syndrome, the triad of insulin resistance, dyslipidemia and arterial hypertension^8^. Adipose tissue (AT) accumulation predominantly occurs in two compartments, the subcutaneous (SAT) and the visceral (VAT) one. SAT is located between the skin and superficial muscles while VAT is found between the abdominal organs. This distinction is of clinical relevance because an excessive VAT amount (relative to total AT volume, TAT) constitutes the metabolically relevant (‘unhealthy’) type of overweight because of its significant association with the metabolic syndrome^8^.

There are different ways to analyze body composition and adipose tissue amounts, such as bioelectrical impedance analysis (BIA), dual-energy X-ray absorptiometry (DEXA), computed tomography (CT) and magnetic resonance imaging (MRI)^9^. Among them, CT and MRI provide true three-dimensional (3D) information and allow for proper segmentation and distinction between all fat compartments. While CT is the more common method for diagnostic imaging, MRI does not expose the body to ionizing radiation.

Tissue segmentation in radiological images will eventually rely on fast and fully automated methods with minimal user interaction^10^. Their development, however, will likely involve an interactive segmentation tool with reviewing and editing options. Over the years, a variety of software tools have been deliberately used for the segmentation of adipose tissue, among them sliceOmatic (Tomovision, Magog, QC, Canada), Osirix^11^, CoreSlicer^12^, ITK-SNAP^13^, MIPAV^14^, 3D Slicer^15^ or ImageJ^16^. Each of them has specific advantages (widely used, flexible or open source) and disadvantages (inter-reader variability, proprietary format or costs). Especially for large cohort studies fully automated approaches based on machine learning are established^17,18^ and commercial solutions exist^19^.

Despite an obligation to buy, Matlab (Mathworks, Natick, MA, USA) is popular in scientific research, has a large developer community and provides a runtime environment for the (free) distribution of applications.

Recently, an open-source Matlab framework, DicomFlex^20^, has been proposed that aims to facilitate the development and extension of common processing tools for medical images. It was designed as a compact, easy-to-use, stand-alone solution for (smaller) research sites, where the task of segmentation is not necessarily performed by the most experienced users.

The aim of this work was therefore to develop and validate a semiautomatic tool for multi-slice MRI segmentation of SAT and VAT amounts under that framework (DicomFlex) using MRI data from study patients with obesity. Our hypothesis was that such a tool is as accurate as an established reference method but – considering the long times for volumetric fat segmentation – substantially faster.

## Materials and methods

### Study population

The retrospective study was carried out within the Integrated Research and Treatment Center AdiosityDiseases of Leipzig University Medicine, Leipzig, Germany, and analysis was approved by the Institutional Review Board (IRB) of the corresponding Faculty of Medicine (reference numbers 283/11-ff, 284/10-ff). Informed consent was obtained from all participants. All methods were carried out in accordance with the Decalaration of Helsinki. 20 patients (25–63 years old) with an average BMI of 36,8 kg/m2 (range: 28,3–58,8) were included. Subcutaneous adipose tissue (SAT) and visceral adipose tissue (VAT) were segmented on axial MRI slices acquired between pelvic floor and diaphragm. In general data was anonymized during the underlying clinical trial. Two radiologist checked MR scans for incidental findings. The readers had access to imaging data, gender and BMI during the initial segmentation on a per-day basis in the 7–10 days after the MRI scan.

## Magnetic resonance imaging

All subjects were examined in supine position in a 1.5T MRI system (Achieva XR, Philips Healthcare, Best, Netherlands). In-phase and opposed-phase images were acquired with a 2D double-echo (TE1: 2.3 ms, TE2: 4.6 ms) spoiled gradient-echo sequence using the integrated quadrature body coil as receive coil. Other sequence parameters were repetition time = 112 ms, flip angle = 70°, slice thickness = 10.0 mm, interslice gap = 0.5 mm, 5 slices per breath-hold, field of view = 530 mm × 530 mm, acquisition matrix size = 216 × 177 and reconstruction matrix size = 480 × 480. Data were acquired in two contiguous stacks aiming to include the whole abdomen between pelvic floor and diaphragm. A breath-hold technique was used to reduce motion artefacts and total acquisition time was 10 × 13 s plus breathing interval times.

## Image analysis

MRI data were analyzed on a standard PC equipped with a quad-core CPU (Intel Core i7-3770, 3.4 GHz) and 16 GB RAM running 64-bit Windows. The semiautomatic SAT/VAT segmentation was designed under the Dicomflex framework^20^ using Matlab version 9.1 and related toolboxes.

**Fig. 1:**
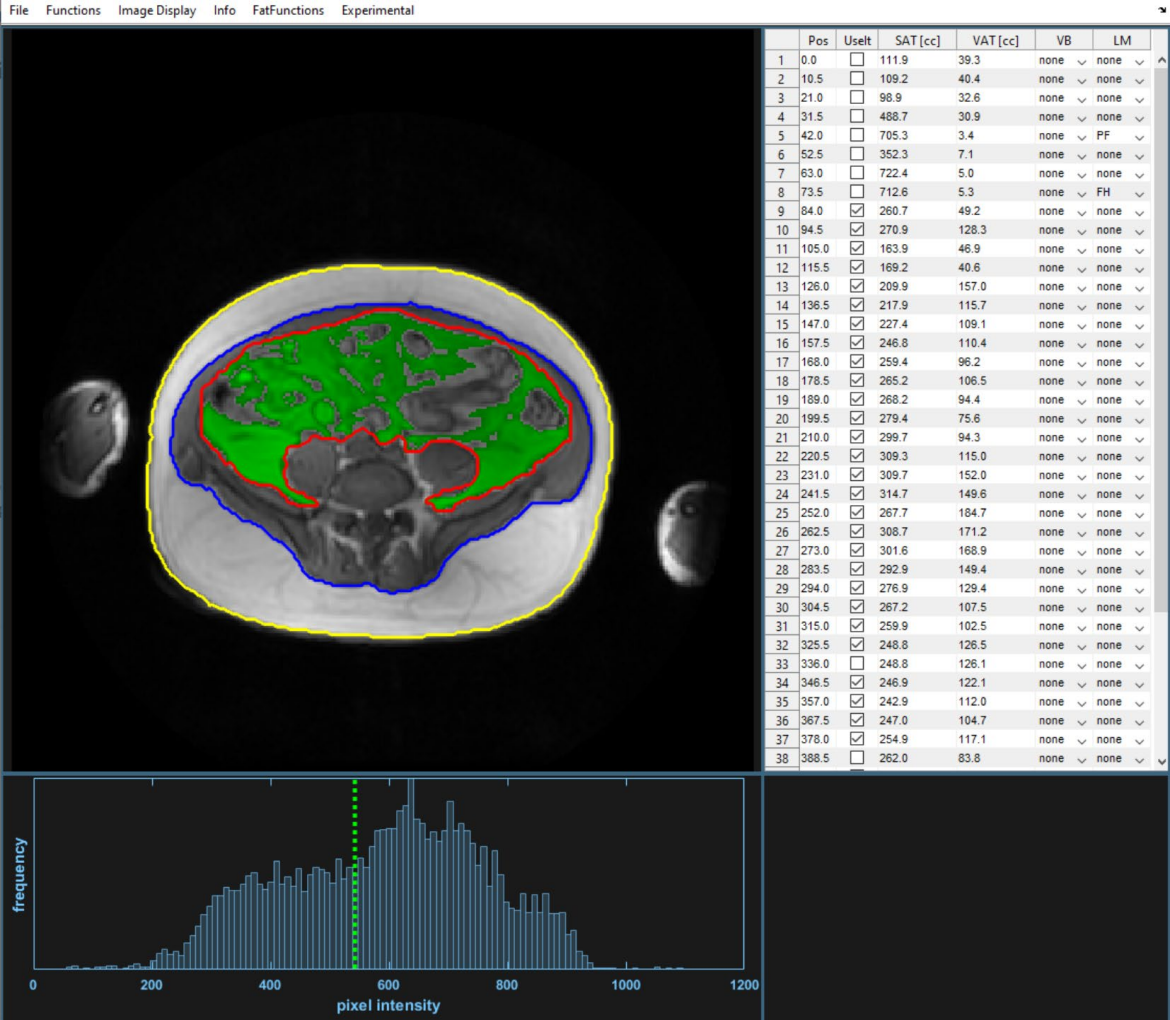
Graphical user interface of stand-alone tool (segfatMR) for multi-slice segmentation of SAT and VAT amounts. After automatic segmentation, the user has the opportunity to review and graphically adjust the boundaries after selecting them by key press (1–3 for outer SAT, inner SAT and VAT, respectively). Unlike the SAT boundary, the VAT boundary is a crude one and fat quantification uses a histogram-based threshold (green dashed vertical line) to separate signal intensities from fat or lean tissues (resulting VAT areas overlaid in green). The interactive table summarizes all results and allows the user to link slices to positions of vertebral bodies (column VB) or landmarks (LM), such as pelvic floor (PF) or femoral heads (FH).

**Fig. 2:**
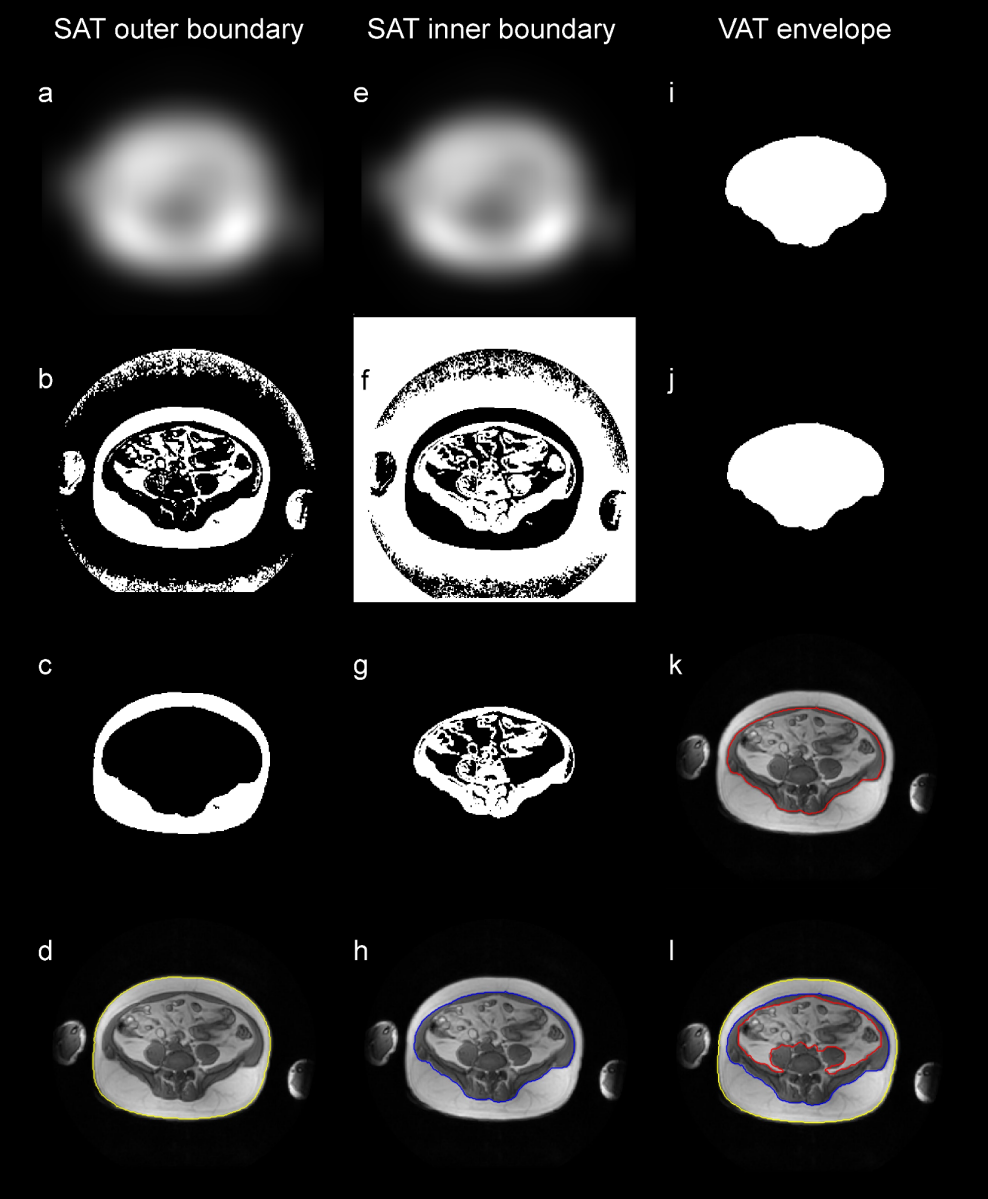
Main processing steps for computation of refined outer SAT (**a-d**, left column), refined inner SAT (**e-h**, middle column) and crude (envelope) VAT boundaries (**i-l**, right column). (**a**) adaptive threshold mask of MR image using *adaptthresh* (Matlab function) with filter size 19 × 19. (**b**) binarized MR image using *imbinarize* and mask from a. Resulting white areas primarily reflect adipose tissue but artefacts are present as well, most notably, at the bottom of the image. (**c**) largest connected white-pixel region from b determined using *regionprops* is considered as SAT. Further criteria were implemented for consistency, for example, the ratio of the principal diameters of considered regions. (**d**) resulting outer (yellow) SAT contour computed using *bwboundaries* on filled mask (not shown here) and overlaid on original MR image. (**e**) adaptive threshold mask with 17 × 17 filter used for binarization and (**f**) resulting binary mask (inverted). (**g**) largest connected region of mask f after multiplication with filled mask for outer SAT contour. (**h**) resulting inner (blue) SAT contour. (**i**) filled mask for inner SAT contour after evaluation of different threshold masks and quantitative criteria (region size and eccentricity). (**j**) eroded mask from i and (**k**) resulting envelope (red) boundary for VAT. (**l**) MR image annotated with all boundaries.

The segfatMR application was realized under a new Dicomflex *cComputeapp* class (*cCompute_segfatMR*), which includes app-specific properties and quantification methods based on in-phase MR images (available in standard Dicom format). The automatic part of the segmentation method (*mAutoSegment*) generates a refined outer (blue) and inner subcutaneous boundary (yellow) and a crude visceral envelope (red), as shown in Fig. 1. Processing details are given in Fig. 2. The last step saves the boundary results as a boundary object, which is a property of the *cCompute_segfatMR* class. The software is part of the existing software framework that is freely *accessible in a public repository as a compiled and packaged MATLAB application. More details on the a*lgorithm and a corresponding documentation are provided on GitHub under https://github.com/Stangeroll/Dicomflex2).

Two independent and experienced users segmented the SAT and VAT amounts using the new (segfatMR) as well as the reference method (sliceOmatic). Times were recorded between the start of the respective application and the final save operation of the segmentation results. In short, segfatMR processing consisted of selecting and loading the images, setting two landmarks (diaphragm, pelvic floor), automatic segmentation and final manual adjustments of the SAT/VAT contours and VAT thresholds where needed. For sliceOmatic, SAT and VAT image regions were independently defined by a region growing algorithm and then refined by a number of standard editing functions (tag colour, delete, lock and surface/volume).

### Statistical analysis

**Fig. 3:**
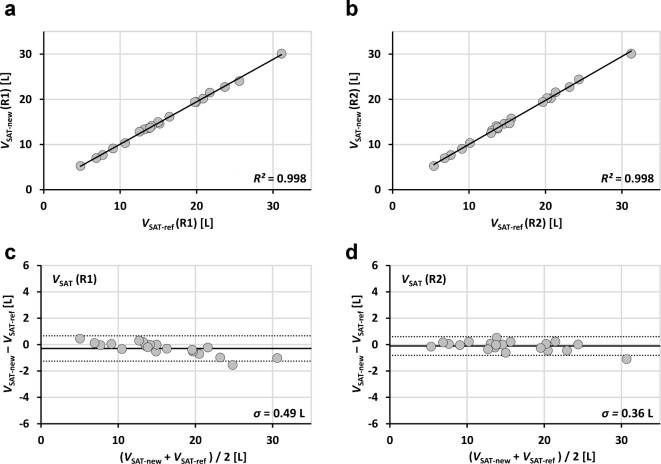
Linear regression results (**a**,**b**) and Bland-Altman plots (**c**,**d**) of abdominopelvic SAT volumes (20 patients) segmented with new (segfatMR) and reference method (sliceOmatic) for two independent readers (R1: **a**, **c** and R2;**b**,**d**).

**Fig. 4:**
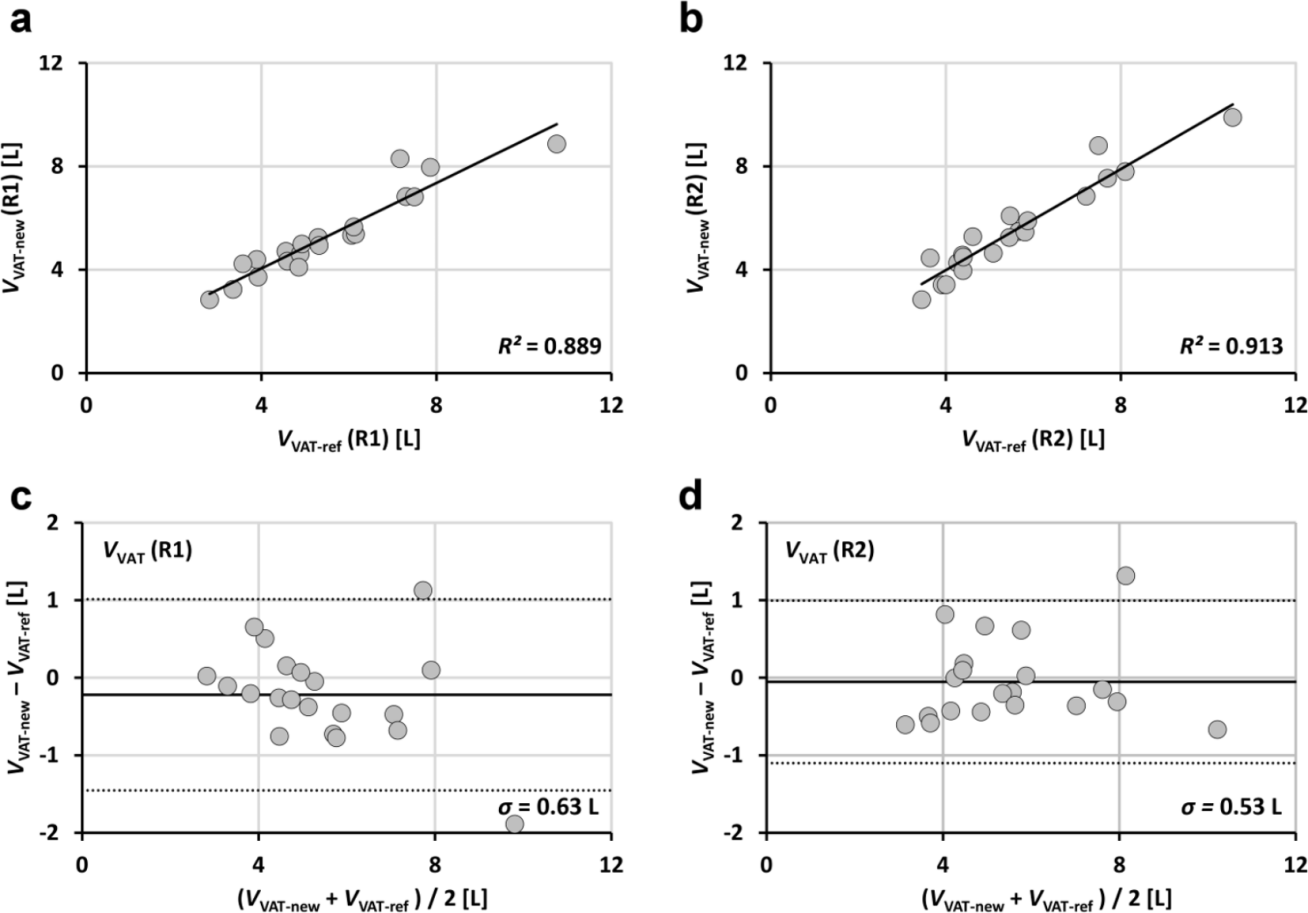
Linear regression results (**a**,**b**) and Bland-Altman plots (**c**,**d**) of total VAT volumes (20 patients) segmented with new (segfatMR) and reference method (sliceOmatic) for two independent readers (R1:**a**,**c** and R2;**b**,**d**).

**Fig. 5:**
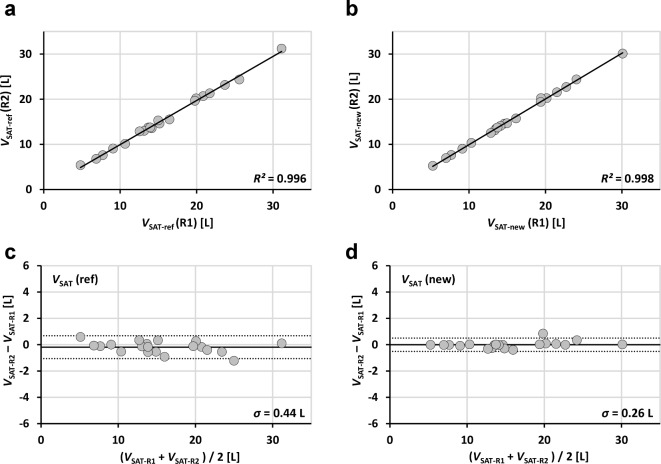
Linear regression results (**a**,**b**) and Bland-Altman plots (**c**,**d**) of abdominopelvic SAT volumes (20 patients) segmented by two readers (R1 and R2) for two methods (ref: **a**,**c** and new **b**,**d**).

Linear regressions were performed between applications and between readers and results are reported as coefficients of determination *R*^2^, the square of Pearson correlation coefficient *r* (Figs. 3, 4 and 5; Table 1). Analyses were performed with SPSS (Version 27, IBM Inc., Armonk, NY, USA). Bland-Altman (BA) analyses are even more appropriate and give the bias and level of confidence for all comparisons. Processing times are shown in boxplot format for both readers and both methods (Fig. 7). Statistical differences were tested at an error level of 0.05.

**Table 1:** Summary of linear regression and Bland–Altman analyses.

Tissue	Reader	Eq. between methods	*R* ^2^_adj_	*σ*_BA_ [L]	Δ_BA_ [L]	*P* (∆_BA_)
SAT	R1	V_new_ = 0.939 ∙ V_ref_ + 0.670 L	0.998	0.49	-0.29	< 0.05 *
	R2	V_new_ = 0.972 ∙ V_ref_ + 0.331 L	0.998	0.36	-0.11	0.206
VAT	R1	V_new_ = 0.826 ∙ V_ref_ + 0.746 L	0.889	0.63	-0.22	0.134
	R2	V_new_ = 0.977 ∙ V_ref_ + 0.074 L	0.913	0.53	-0.05	0.656

Tissue	Method	Eq. between readers	*R* ^2^_adj_	*σ*_BA_ [L]	Δ_BA_ [L]	*P* (*σ*_BA_)
SAT	*ref*	V_R2_ = 0.977 ∙ V_R1_ + 0.176 L	0.996	0.44	-0.19	0.072
	*new*	V_R2_ = 1.013 ∙ V_R1_ – 0.199 L	0.998	0.26	0.00	0.958
VAT	*ref*	V_R2_ = 0.908 ∙ V_R1_ + 0.541 L	0.883	0.63	0.03	0.836
	*new*	V_R2_ = 1.100 ∙ V_R1_ – 0.336 L	0.954	0.42	0.20	0.051

*ref/new*: reference/new method (sliceOmatic/segfatMR); *R*^2^_adj._ adjusted R square.

*σ*_BA_/Δ_BA_/*P* (Δ_BA_): standard deviation/mean (bias) of volume differences [L], *P* value for bias.

## Results

**Fig. 6:**
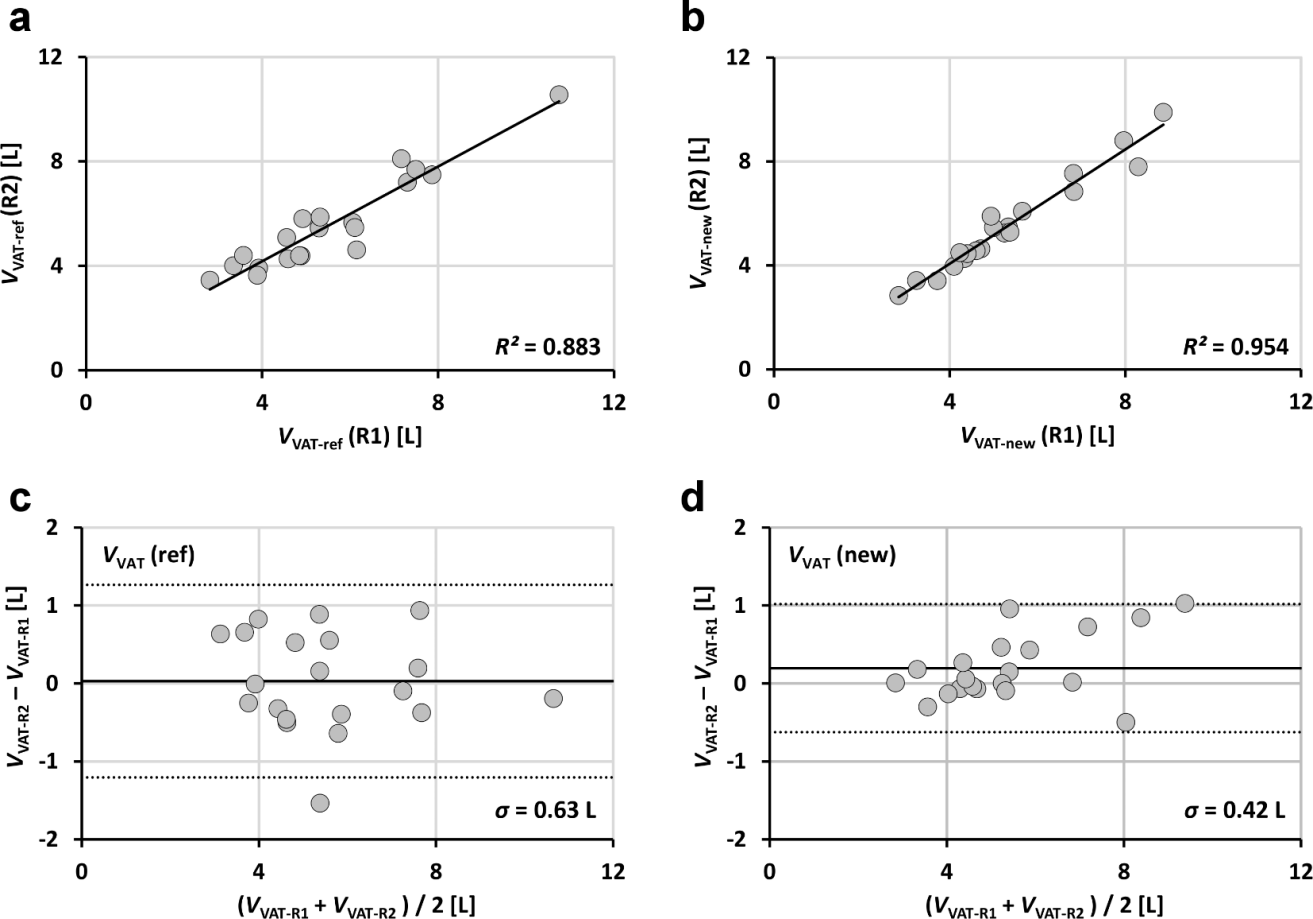
Linear regression results (**a**,**b**) and Bland-Altman plots (**c**,**d**) of total VAT volumes (20 patients) segmented by two readers (R1 and R2) for two methods (ref:**a**,**c** and new **b**,**d**).

Figures 3, 4, 5 and 6 show the regression lines over the data points (SAT and VAT volumes) for all patients with the corresponding BA plots and for all comparisons between methods and readers. Plots are annotated with coefficients of determination *R*^2^ (adjusted values reported by SPSS) and standard deviations *σ*_BA_ of the respective differences.

Agreement of abdominopelvic SAT volumes between methods (Fig. 3) is excellent (*R*^2^ > 0.99 for both readers R1 and R2) with *σ*_BA_ values of 0.49 L (R1) and 0.36 L (R2). Figure 4 shows the corresponding data for VAT with lower *R*^2^ (around 0.90) and higher *σ*_BA_ values (0.63/0.53 L). Figure 5 shows the interreader agreement of adipose tissue segmentation which is very high for SAT and for both methods (*R*^2^ > 0.99, *σ*_BA_ = 0.44/0.26 L). Figure 6 shows the interreader plots for total VAT volumes. Despite being lower than for SAT, *R*^2^ values are still high (> 0.88) with a notably higher interreader agreement for the new method (*R*^2^: 0.954 vs. 0.883; *σ*_BA_: 0.42 L vs. 0.63 L), which is also visually apparent. All BA bias values were not significant but between methods for SAT segmentation by R1. Table 1 summarizes the numerical results for all linear regression and BA analyses (between methods and readers).

**Fig. 7:**
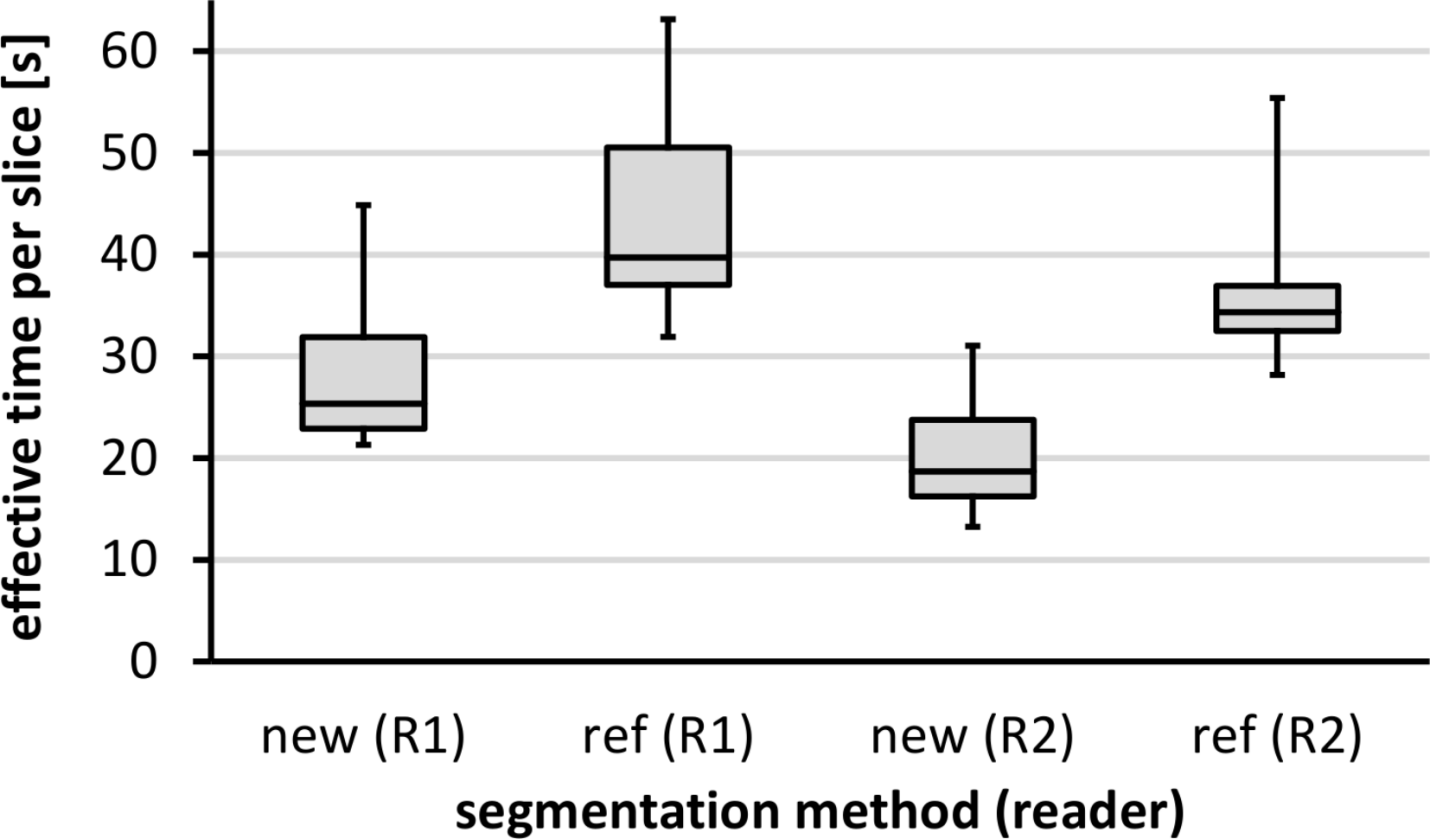
Boxplot of effective processing times (in seconds) per slice for fat segmentation with the new (fatSeg) and the reference method (sliceOmatic) and both readers (R1 and R2). Boxes mark quartiles Q1-Q3 and whiskers minimum and maximum times.

Whole abdominal coverage involved 38.2 ± 3.2 (29–46) slices. Figure 7 shows boxplots of the effective processing times per slice for both methods and readers. There is a highly significant (*p* < 0.001) and, more importantly, notable reduction in processing time for both readers (R1: from 40 to 25 s, R2: from 34 to 19 s) with the new method (segfatMR) which translates to a time saving on the order of 10 min for the segmentation of all abdominal slices here. The lowest median time for abdominal fat segmentation was 11.4 min (R2).

## Discussion

Efforts to quantify adipose tissue amounts by imaging have focused on the identification of surrogate measures or landmark slices mainly because interactive processing of all abdominopelvic slices (typically between diaphragm and pubic symphysis) is laborious. The segfatMR tool may facilitate analyses or actually enable them. Despite a substantial reduction in analysis time, agreement with the reference and between readers was high. Reader interaction was limited to visual inspection of the computed AT contours (corrected by simply drawing new segments where needed) and of the overlaid VAT amounts (corrected by adjusting the threshold for tissue assignment). Commercial tools usually provide a larger range of options, but segmentation may involve annotations across the entire slice and a choice of specific display and processing functions.

Despite some additional efforts and time, a supervised approach will also work with highly variable anatomical distribution patterns and body shapes. Imaging artefacts from breathing or organ motion, among others, can be reliably identified and corrected for. Another aspect is the detection of incidental findings. Moreover, the segfatMR tool and other Dicomflex applications may be easily modified or extended.

MRI examinations are more complex and less common than CT scans, take longer and have special contraindications for patients with devices or implants. The confined geometry of an MRI system poses a challenge for patients with claustrophobia or overweight. AT segmentation requires adequate training in the reading of MR images. MRI signal intensities may also vary across the field of view for technical reasons like magnetic field inhomogeneities. This work has a number of limitations. Patients were selected from a bariatric cohort with higher VAT amounts which are easier to distinguish from surrounding structures like bowel, pancreas or urinary bladder. This bias might also explain the low inter-rater variability. A second limitation is the reference standard, which only relies on the assessment of two readers. The third limitation is the use of specific input data, in-phase images of a two-point Dixon sequence, which has been agreed upon in the original clinical study protocol.

Segmentation of SAT is less demanding, both visually (reader) as well as technically (algorithm). Patients with lipodystrophy are an exception but our experience is limited here. The tool also computes a histogram threshold for each slice that distinguishes fat from lean tissue and can be adjusted manually (slider). This threshold applies to tissues within the entire VAT contour and may therefore require a threshold adjustment (towards higher values) that deliberately excludes minor (true) VAT amounts to make up for falsely included fat amounts in non-VAT regions, typically a pronounced fatty contamination in the bowels. In contrast, sliceOmatic allows for a dedicated, but also more time-consuming, focal editing of VAT amounts. Despite two slightly different approaches, our analysis revealed no significant bias between the respective VAT volumes. So far, our analysis has relied on MRI data from a two-point Dixon sequence, which is typically not part of the routine protocol. One of the main reasons for implementation under a dedicated software framework (DicomFlex), however, was the ability to flexibly adjust to different input data (standard T1-weighted sequence). In terms of time investment, the presented semiautomatic fat segmentation lies between traditional, essentially manual (interactive) and fully automatic approaches.

Segmentation tasks will likely benefit from the ongoing progress in deep learning (DL) techniques. While analysis times for whole-abdominal datasets are expected to be much shorter, DL tools are not widely available and might be less accurate for cases further away from the datasets used for their training. Large, carefully annotated datasets – and powerful CPU or GPU engines – are required to reach the accuracy level of human experts. The question to what extent DL image processing may generate artifacts or obscure actual features – also depending on the training datasets – is currently not fully answered.

Research on automatic segmentation was proposed early^21^, even for obese patients^17^. There is preliminary evidence that therapy-induced weight loss can also be quantified in an automated manner^22^, but smaller changes in volumes of adipose tissue and organs very likely require a direct manual inspection of the images.

As more advanced multi-echo Dixon techniques become routinely available, proton density fat fraction (PDFF) data might be more reliable for the design of DL-based adipose tissue segmentation. Such an approach has recently been used for whole-body (neck-to-knee) MRI measurements in 20 volunteers. A combination of segmentation techniques for different tissues might pave the way for a comprehensive and rapid analysis of the body composition. Until then, semiautomatic tools like the one presented will still be indispensable (a) to obtain highly accurate results in a reasonable amount of time, (b) to provide training data for deep-learning approaches or (c) in cases where visual inspection by a radiologist is required by the study protocol (e.g., to check for incidental findings).

The tool is significantly faster and less laborious than common manual methods like the commercial reference used here (sliceOmatic). With an average processing time over an entire abdominopelvic dataset of around 20 s per slice on current standard hardware, it appears to be suitable for small to moderately sized quantification tasks aiming at narrower limits of agreement than simple single-slice estimates.

In conclusion, we have presented and validated a semiautomatic software tool (segfatMR) for the segmentation of different abdominal adipose tissue amounts between diaphragm and pelvic floor. The software is easy to use, requires basic anatomical knowledge only and may generate the training datasets required for automated segmentation approaches. Hardware requirements are relatively low and no prior information or validation is needed for particular cohorts or questions. Further code adaptation and application deployment are facilitated by our open-source design.

## Data Availability

The datasets generated and/or analyzed during the current study are not publicly available due to local data safety restrictions but are available from the corresponding author on reasonable request.
